# Highly-efficient growth of cobalt nanostructures using focused ion beam induced deposition under cryogenic conditions: application to electrical contacts on graphene, magnetism and hard masking†

**DOI:** 10.1039/d1na00580d

**Published:** 2021-08-25

**Authors:** Alba Salvador-Porroche, Soraya Sangiao, César Magén, Mariano Barrado, Patrick Philipp, Daria Belotcerkovtceva, M. Venkata Kamalakar, Pilar Cea, José María De Teresa

**Affiliations:** Instituto de Nanociencia y Materiales de Aragón (INMA), CSIC-Universidad de Zaragoza 50009 Zaragoza Spain deteresa@unizar.es j.deteresa@csic.es; Laboratorio de Microscopías Avanzadas (LMA), Universidad de Zaragoza 50018 Zaragoza Spain; Advanced Instrumentation for Nano-Analytics (AINA), MRT Department, Luxembourg Institute of Science and Technology (LIST) 41 rue du Brill 4422 Belvaux Luxembourg; Department of Physics and Astronomy, Uppsala University Box 516 SE-751 20 Uppsala Sweden

## Abstract

Emergent technologies are required in the field of nanoelectronics for improved contacts and interconnects at nano and micro-scale. In this work, we report a highly-efficient nanolithography process for the growth of cobalt nanostructures requiring an ultra-low charge dose (15 μC cm^−2^, unprecedented in single-step charge-based nanopatterning). This resist-free process consists in the condensation of a ∼28 nm-thick Co_2_(CO)_8_ layer on a substrate held at −100 °C, its irradiation with a Ga^+^ focused ion beam, and substrate heating up to room temperature. The resulting cobalt-based deposits exhibit sub-100 nm lateral resolution, display metallic behaviour (room-temperature resistivity of 200 μΩ cm), present ferromagnetic properties (magnetization at room temperature of 400 emu cm^−3^) and can be grown in large areas. To put these results in perspective, similar properties can be achieved by room-temperature focused ion beam induced deposition and the same precursor only if a 2 × 10^3^ times higher charge dose is used. We demonstrate the application of such an ultra-fast growth process to directly create electrical contacts onto graphene ribbons, opening the route for a broad application of this technology to any 2D material. In addition, the application of these cryo-deposits for hard masking is demonstrated, confirming its structural functionality.

## Abstract 2

Direct electrical contacts (no resist is involved) have been grown on graphene ribbons by the Cryo-FIBID technique for the first time, opening the route for a wider use on other 2D materials.

## Graphical abstract

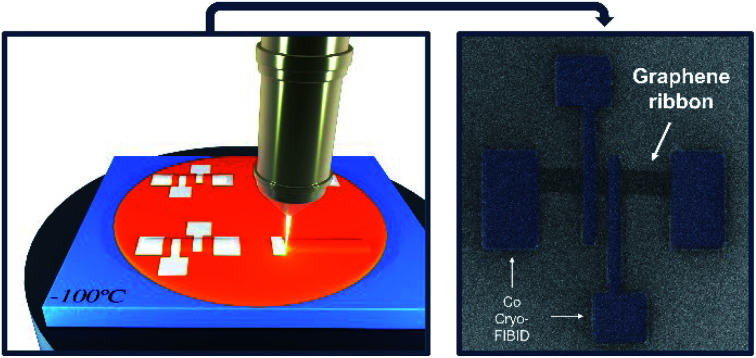

## 1. Introduction

The fabrication of functional nanostructures by the top-down approach generally involves the use of thin-film deposition and nanolithography techniques.^1^ The great advantage of this approach is the creation of multiple nanostructures on a single substrate with tailored dimensions. The fabrication of metallic nanostructures (used in the semiconductor industry, nanoelectronics, sensors, *etc.*) and magnetic nanostructures (used in data storage, spintronics, *etc.*) mostly follows this approach.^2,3^ However, the use of sacrificial resist layers^4^ and the several steps normally required, such as in optical lithography,^5^ electron beam lithography,^6^ nanoimprint lithography,^7^*etc.* often imply long processing times and technical challenges. As a consequence, whereas resist-based nanolithography is often convenient, a growing interest exists in resist-free nanolithography techniques (based on focused charged beams, atomic force microscopy, shadow mask, *etc.*), which provide the capability for fast prototyping,^8^ 3D patterning,^9^ very high lateral resolution,^10,11^ the patterning on delicate substrates and cantilevers,^12,13^*etc.* However, such resist-free processing is generally slow, limiting its use to a few applications such as circuit edit,^14^ mask repair,^15^ and lamellae preparation.^16^ Efforts towards efficient and faster resist-free methods could bring new prospects and increase its impact, contributing to devise new ways for custom-tailored patterning.

In this work, we focus our attention on the resist-free growth of functional nanostructures by means of focused ion beam induced deposition (FIBID).^17^ FIBID consists in the adsorption of precursor molecules provided by a gas injection system (GIS) on the substrate surface, which are dissociated by a focused ion beam (typically based on Ga^+^), producing the growth of a deposit. In this technique, generally performed at room temperature, a single monolayer of precursor molecules is adsorbed on the substrate surface and most of the energy provided by the focused ion beam is not used for precursor dissociation, making the process inefficient. As a consequence, the growth rate is low (for Pt-based deposits, it equals 0.5 μm^3^ nC^−1^)^18^ and the use of this technique is limited to the niche applications previously mentioned (circuit edit, mask repair, lamellae preparation and prototyping).^1^ It is worth noting that the sister technique FEBID (E stands for electrons instead of ions) exhibits even lower growth rates.^18^

Interestingly, the growth rate in FIBID can be increased several orders of magnitude if the precursor molecules form a thick layer where most of the beam energy is absorbed and efficiently used for molecule dissociation. This can be achieved through cooling the substrate below the precursor condensation temperature.^19–24^ This Cryo-FIBID approach holds an enormous potential for rapid patterning of metallic micro/nano-deposits. For example, optimized W–C cryo-deposits with a resistivity of 800 μΩ cm were obtained using 50 μC cm^−2^ Ga^+^ dose and applied as electrical contacts to measure semiconducting nanowires. Certainly, nanolithography under cryogenic conditions is a promising route to carry out certain patterning processes, with great advantages over the traditional nanopatterning techniques, and ice lithography is one of such examples.^25^

Here we demonstrate the ultra-fast growth by Cryo-FIBID of micro/nano-deposits bearing metallic and magnetic functionality. For that, we use the Co_2_(CO)_8_ precursor material, which has shown excellent metallic and magnetic properties when grown by FIBID at room temperature, albeit with a low growth rate. *Via* cryogenic FIBID nanopatterning, we have found that a very low Ga^+^ dose of only 15 μC cm^−2^ (two thousand times lower than the same process at room temperature) is sufficient to achieve a metallic content close to 60% (in atomic percent), giving rise to deposits exhibiting ferromagnetic behaviour and metallic conduction with a resistivity value as low as 200 μΩ cm at room temperature. As a proof of concept, we apply Cryo-FIBID of cobalt to successfully make electrical connections to graphene monolayer (without destroying graphene), create magnetic nanostructures and further demonstrate its application as a hard mask in an Ar^+^ milling process. Thus, the process developed here represents a unique novel platform for direct nanolithography, encompassing potential applications in the semiconductor industry, nanoelectronics, sensing, nanomagnetism, and nanotechnology.

## 2. Experimental section

### 2.1. Fabrication of cobalt-based deposits

**Fig. 1:**
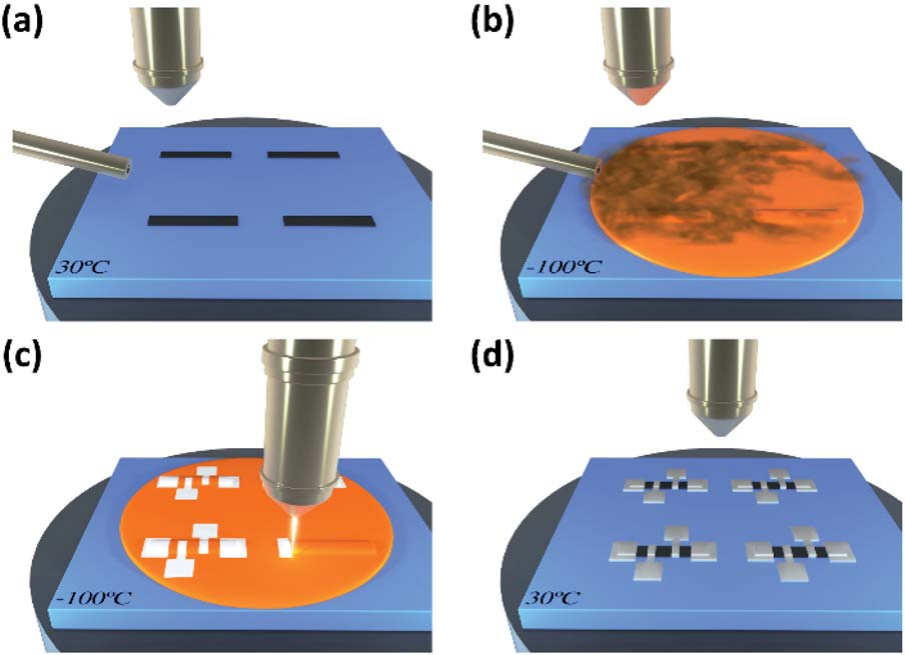
Cryo-FIBID process used to grow electrical contacts on 2D materials (*e.g.*, graphene). (a) Deposition of the material on a Si/SiO_2_ substrate. (b) Aperture of GIS on the cooled substrate, giving rise to a precursor condensed layer. (c) Focused ion beam irradiates the condensed layer with the wanted pattern. (d) Substrate heating up to 30 °C producing the sublimation of the non-irradiated condensed layer.

Si substrates were used for the growth optimization process and magnetic measurements, whilst Si substrates with a thermally grown 285 nm-thick SiO_2_ layer were used for electrical measurements. In order to test the growth of electrical contacts onto graphene, we employed graphene ribbons of chemical vapor deposited (CVD) graphene that are patterned by optical lithography and oxygen plasma etching. The growth of cobalt deposits by Cryo-FIBID technique was carried out on a Nova NanoLab 200 Dual Beam instrument (FEI Company) equipped with a cryo-setup module (Quorum Technologies) that provides substrate temperatures down to −155 ± 5 °C. For cobalt deposition, a Ga^+^ focused ion beam column in combination with an individual GIS of Co_2_(CO)_8_ precursor molecules, were used. The process consists in cooling the substrate from room temperature to −100 °C in less than 10 minutes, opening the GIS valve for a few minutes, Ga^+^ irradiation with the appropriate dose, and substrate heating up to 30 °C in less than 5 minutes (staying at that temperature for 15 minutes). The ion irradiation dose was varied in the range from 6 to 50 μC cm^−2^. In order to illustrate the process, we sketch in Fig. 1 how one should proceed to grow electrical contacts on nano-objects based on the Cryo-FIBID technique. The steps shown in Fig. 1 are the ones followed in the present work for the direct growth of cobalt electrical contacts on graphene ribbons.

### 2.2. Characterization techniques

#### Electrical measurements

To obtain the current-*versus*-voltage curves, four-probe electrical measurements were performed using electrical microprobes (Kleindiek Nanotechnik GmbH), and a 6221 DC current source/2182A nanovoltmeter (Keithley Instruments). These measurements consist in applying a current sweep through the two outer electrical contacts, while the voltage drop across the two inner contact pads is measured.

#### Magnetic measurements

A large sample (∼0.62 mm^2^) formed by four square deposits was grown for magnetic characterization. The measurements were carried out in an MPMS-XL magnetometer (Quantum Design), which is based on a superconducting quantum interference device (SQUID) that can measure very low magnetic signals. To improve the overall sensitivity and noise reduction for DC magnetization measurements, the Reciprocating Sample Option (RSO) was used.

#### Compositional analysis

A large number of samples were compositionally analysed by energy-dispersive X-ray spectroscopy (EDS) using an Inspect F-50 scanning electron microscope (FEI Company) and equipped with an INCA 350 detector (Oxford Instruments). Additionally, chemical analyses were performed by electron energy loss spectroscopy (EELS) in cross-sectional specimens by Scanning Transmission Electron Microscopy (STEM) in a Titan Low Base microscope (FEI Company). Annular dark field (ADF) images were obtained at 300 keV in STEM mode.

### 2.3. Numerical simulations

Insights into the implantation and sample modification by the Ga^+^ beam during the Cryo-FIBID process have been obtained by Monte Carlo simulations based on the binary collision approximation using the SDTrimSP software.^26^ The latter allows for the modelling of changes in sample composition and thickness with primary ion dose. In this work, the interatomic interactions are described by the KrC potential, and the Oen-Robinson model is used for the electronic stopping. Integration is carried out with the Gauss-Mehler method with sixteen pivots. The surface binding energy is calculated using the equation sbe(*i*, *j*) = 0.5 × (Es_*i*_ + Es_*j*_), where sbe is the surface binding energy of the current target and Es_*i*_ is the atomic surface binding energy of the chemical species *i*. The surface binding energy is calculated for any combination of two species that are used in this work, *e.g.* gallium, silicon, cobalt, carbon and oxygen.^26^ For the different species, the atomic densities have been taken identical to the bulk values. Hence, the density of the precursor layer in the simulations might be above the value in the experiments. As the experimental diffusion coefficients of the different species are not known, this effect has been neglected in the modelling, although SDTrimSP is capable of taking it into account.^4,27^ For volatile species and the desorption of small clusters, this effect might be significant. The default displacement energies coming with the software have been used: 12 eV for gallium, 13 eV for silicon, 22 eV for cobalt, 25 eV for carbon and 0.5 eV for oxygen. The precursor layer has a thickness of 10 to 60 nm, gallium ion bombardment is simulated for an impact energy of 30 keV at normal incidence. The dose has been increased up to a maximum of 56 μC cm^−2^.

## 3. Results and discussion

### 3.1. Simulations of the interaction of the ion beam with the cobalt condensed layer

**Fig. 2:**
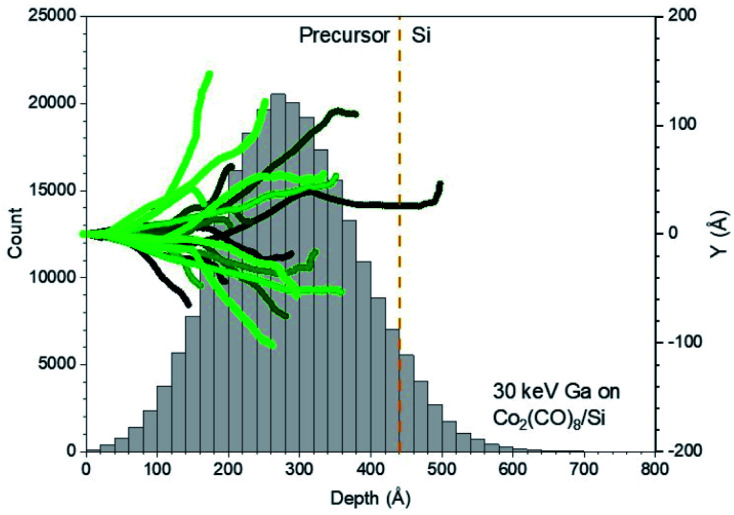
SDTrimSP simulations of 30 keV implanted Ga^+^ ions on a 44 nm-thick condensed layer of Co_2_(CO)_8_. Left axis represents the distribution of implantation depths. Right axis represents the different trajectories with different shades of green.

The appropriate thickness for deposits grown by Cryo-FIBID technique is given by the ion penetration length. If the ion penetration length is too short to decompose the whole precursor condensed layer, this layer could lift-off during the heating step. On the contrary, if the ion penetration length is higher than the precursor condensed layer, a considerable part of the ion energy will be delivered to the substrate and, consequently, a higher ion dose would be required. Interactions of 30 keV Ga^+^ ions with the Co_2_(CO)_8_ condensed layer were simulated to find out the optimum thickness for the condensate. Fig. 2 shows that the average 30 keV-Ga^+^ penetration length for a 44 nm-thick Co_2_(CO)_8_ condensed layer is 28 nm. For this reason, one of our aims is to control the relevant parameters to get a Co_2_(CO)_8_ condensed layer with thickness in the 28 ± 8 nm range (see section 3.2). Furthermore, individual trajectories under 30 keV Ga^+^ bombardment with different scenarios of the types of collision cascades are illustrated in Fig. S1 in ESI.†

### 3.2. Optimization of growth parameters for the formation of a homogeneous Co_2_(CO)_8_ condensed layer

As mentioned in the previous section, one of the main goals in this work is to optimize the growth parameters to get the condensed layer with a thickness in the 20–36 nm range. These cobalt deposits were grown in 60 μm *×* 5 μm areas on a Si substrate and using a 30 keV Ga^+^ beam. The following growth parameters were fixed: the substrate was cooled down to −100 °C and the distance between the substrate and the GIS was chosen to be 10 mm. The thickness of the condensed layer was controlled through the opening time of the GIS. The GIS valve was opened for 60, 90, 120, 140, and 180 s in five different experiments. In each experiment, ion doses in the 0.5 to 200 μC cm^−2^ range were delivered using a beam current of 1 pA and the average deposit thickness for each aperture time was determined. The trend between the thickness of the condensed layer after irradiation and the aperture time of GIS proved to be lineal (see Fig. S2 in ESI†). An aperture time of 140 s was chosen for the next experiments to obtain an irradiated condensed layer of about 28 nm in thickness. The deposits grown with doses lower than 6 μC cm^−2^ were not continuous owing to the fact that the irradiation dose was not able to sufficiently decompose the condensed layer. On the other hand, EDS results showed that the metallic content did not increase for ion doses beyond 50 μC cm^−2^, which led us to focus the study on the 6 μC cm^−2^ to 50 μC cm^−2^ range.

### 3.3. Electrical characterization of cobalt deposits by the four-probe technique

**Fig. 3:**
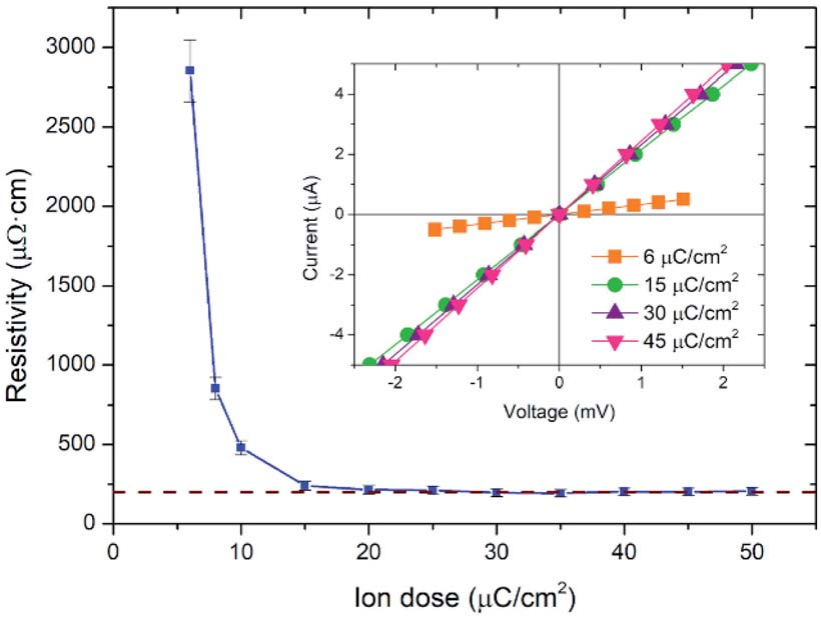
Electrical resistivity of cobalt deposits as a function of the ion dose used for their fabrication by Cryo-FIBID technique. The inset shows *I*–*V* measurements of some selected samples.

Cobalt deposits of ∼600 μm^2^ grown on a Si/SiO_2_ substrate under the optimized conditions were electrically characterized at room temperature by the four-probe technique using electrical microprobes. As it can be observed in the inset of Fig. 3, a linear current-*versus*-voltage (*I*–*V*) dependence exists for the whole range of irradiation doses studied, as expected for an ohmic behaviour. From linear fits to the *I*–*V* data, the electrical resistance was extracted and represented as a function of the ion dose (see Fig. S3 in ESI†). It is observed that the electrical resistance decreases abruptly as a function of the ion dose until 15 μC cm^−2^, with little variation for higher ion doses. After measuring the thickness of the deposits with a profilometer, the electrical resistivity was calculated and represented in Fig. 3. The electrical resistivity decreases as a function of the ion dose until 15 μC cm^−2^, being around 200 μΩ cm for higher doses. The error bar in these experimental results comes from the instrumental error of the profilometer, which is of 3 nm.

Considering the low irradiation time and low electrical resistivity, an ion dose of 15 μC cm^−2^ is considered an optimized value for application of these deposits as electrical contacts. This means a growth rate with a 2 × 10^3^ yield enhancement compared with cobalt deposits grown at room temperature by FIBID,^28^ and with a 10^5^ yield enhancement when compared with cobalt deposits growth at room temperature by FEBID.^29^

**Fig. 4:**
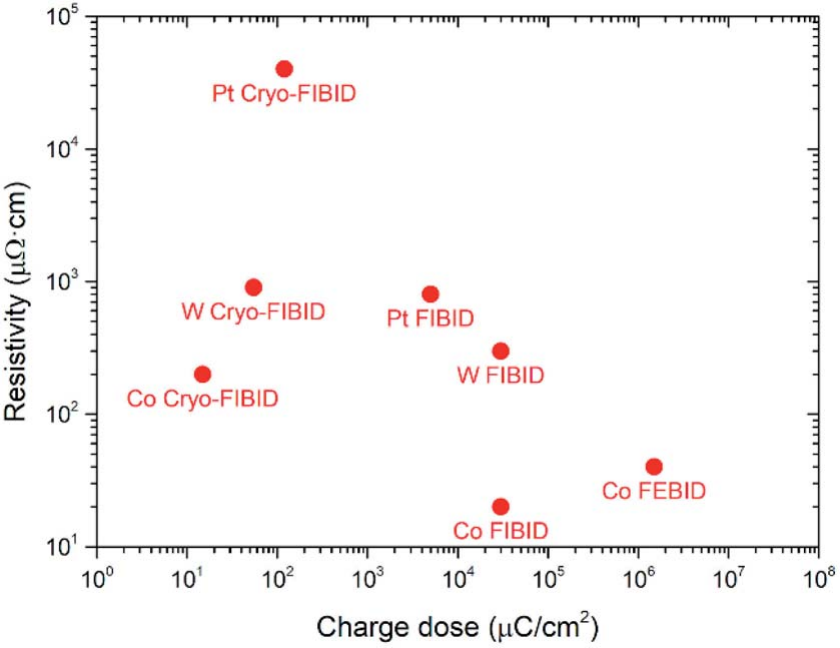
Comparison of different FEBID and FIBID techniques in terms of the optimized dose and its electrical resistivity. The experimental points other than Co Cryo-FIBID have been obtained from ref. 18, 21, 23, 28 and 29.

Fig. 4 shows the comparison of different FEBID and FIBID techniques in relation to the required dose and its electrical resistivity. When Co Cryo-FIBID deposits are compared with other Cryo-FIBID deposits, they turn out to be the least resistive ones, and those that need the lowest dose. The high growth rate and the low electrical resistivity of cobalt deposits grown by Cryo-FIBID are extremely promising for the fast growth of large-area electrical contacts, as will be later shown.

### 3.4. Compositional analysis of cobalt deposits by SEM-EDS and STEM-EELS

First, SEM-EDS experiments were performed in cobalt deposits that had been previously characterized electrically, allowing a direct correlation between their electrical resistivity and the composition. The experiments were carried out using 2 keV primary electrons on 13 μm *×* 7 μm selected areas, from which carbon (C), oxygen (O) and cobalt (Co) were quantified. Table 1 shows the atomic composition of our deposits in the 6 μC cm^−2^ to 50 μC cm^−2^ range. The high cobalt content (50–60% at) agrees with the low electrical resistivities obtained.

**Table 1:** Elemental quantification by EDS of carbon (C), oxygen (O) and cobalt (Co) in cobalt deposits grown by Cryo-FIBID

Ion dose (μC cm^−2^)	C (% at.)	O (% at.)	Co (% at.)
6	21.9 ± 1.8	35.1 ± 1.2	43.0 ± 0.2
8	24.1 ± 1.6	28.3 ± 1.2	47.6 ± 0.2
10	21.6 ± 1.7	24.0 ± 1.3	54.4 ± 0.2
15	19.5 ± 1.7	23.7 ± 1.2	56.8 ± 0.2
20	22.8 ± 1.7	22.6 ± 1.3	54.6 ± 0.2
25	24.6 ± 1.6	23.2 ± 1.3	52.2 ± 0.2
30	21.3 ± 1.8	19.3 ± 1.4	59.4 ± 0.2
35	23.8 ± 1.7	16.7 ± 1.6	59.5 ± 0.2
40	23.6 ± 1.7	17.2 ± 1.5	59.2 ± 0.2
45	24.0 ± 1.6	17.8 ± 1.4	58.2 ± 0.2
50	20.5 ± 1.8	16.3 ± 1.5	63.2 ± 0.2

As expected, a lower metallic content was observed in deposits with doses lower than 10 μC cm^−2^. It is also important to mention that Ga doping is below the detection limits of EDS, contrary to what is observed for other precursors materials used in Cryo-FIBID, in agreement with the very low Ga dose required here.^21,23^

In addition, SDTrimSP simulations were performed for a 44 nm Co_2_(CO)_8_ condensed layer. Fig. S4 in ESI† shows an example of these composition simulations with the case of an ion dose of 50 μC cm^−2^. SDTrimSP simulations were carried out for ion doses in the 5 μC cm^−2^ to 50 μC cm^−2^ range. In all cases, it predicts a cobalt content of 11% at., which is much lower than in the experimental results, with the predicted C and O contents being 44% each. This is explained by the formation of volatile products, such as CO_*x*_, which is not considered in the MC simulations. The maximum concentration for implanted gallium is 0.2%, which agrees with the absence of gallium in the EDS experiments. Moreover, the compositional results obtained by EELS in STEM mode (STEM-EELS) were consistent with EDS results (see Fig. S5 in ESI†).

### 3.5. Magnetic characterization of cobalt deposits by SQUID magnetometry

**Fig. 5:**
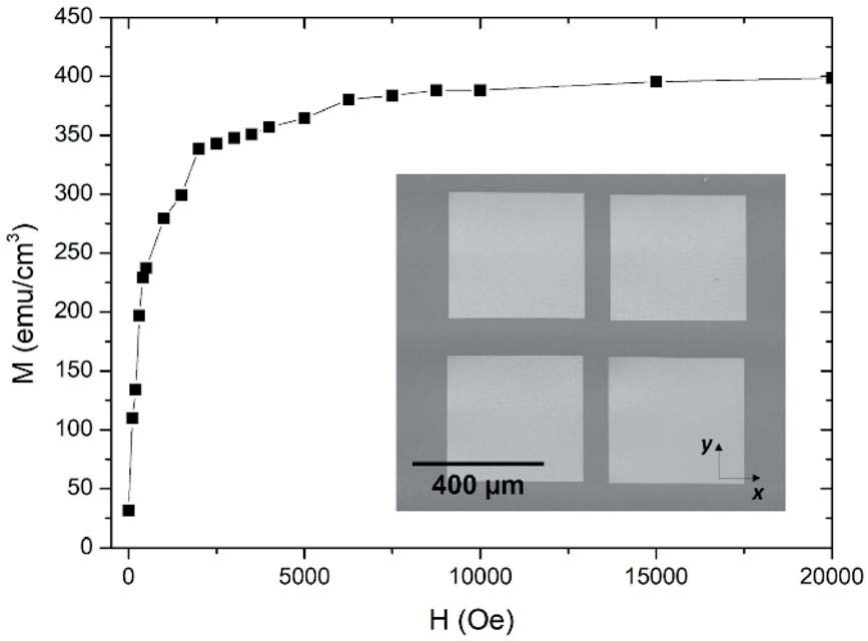
Magnetization of Co Cryo-FIBID deposits with the magnetic field applied along the *X* axis. The inset shows the sample grown under the optimized conditions which was magnetically characterized by SQUID measurements.

For these measurements, four deposits of area ∼400 μm × 400 μm were grown by Cryo-FIBID on a Si substrate under the optimized conditions (ion dose of 15 μC cm^−2^) and using an ion current of 3 nA (see inset in Fig. 5). The thickness of these deposits, as measured by means of a profilometer, resulted to be ∼30 nm. As illustrated in Fig. 5, the magnetization measurements as a function of magnetic field at room temperature indicate that, in magnetic saturation, these cryo-deposits present a magnetization value of 400 emu cm^−3^, which is about one third of the bulk cobalt value. If we compare this result with other cobalt nanostructures, like the nanospheres of Lavenant *et al.*^30^ grown by FEBID (75 ± 5% at. cobalt), we find that their deposits have a magnetization value of 430 ± 80 emu cm^−3^, very similar to ours. However, the increment of 10^5^ in the growth rate of Cryo-FIBID compared to FEBID for the growth of Co deposits is a substantial advantage.^29^

### 3.6. Achievement of high resolution in patterning by Cryo-FIBID

**Fig. 6:**
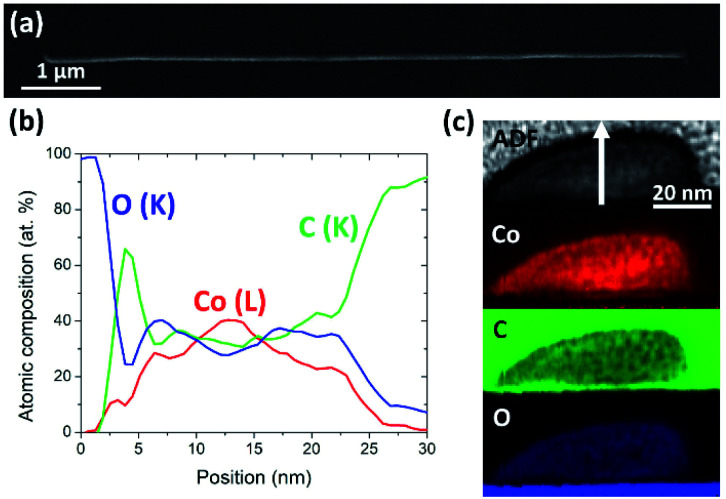
(a) SEM micrograph of a cobalt nanostructure grown using a line pattern with an ion dose of 8 μC cm^−2^. (b) STEM-EELS chemical analysis of the cross-section of a 20 nm-thick cobalt nanostructure, along the white arrow in (c). (c) ADF image and STEM-EELS chemical maps of cobalt, carbon, and oxygen.

As mentioned in the previous sections, Cryo-FIBID technique allows the ultra-fast growth of cobalt deposits with ferromagnetic behavior and metallic conductance. To prove the applicability of these deposits as nanostructures, we have grown cobalt nanowires and studied their resolution and composition by STEM-EELS. Fig. 6a shows a SEM micrograph of a long Co Cryo-FIBID nanowire (∼8 μm) performed with a line patterning in just 14 ms, corresponding to an ion dose of 8 μC cm^−2^. As shown in Fig. 6b, the cross-section of our nanostructure was compositionally analyzed by STEM-EELS, showing a Co content up to 40% at., with a maximum metallic content at the core of the nanostructure. The vertical compositional profile is indicated in the ADF image in Fig. 6c and it is concluded that we can achieve cobalt nanostructures with a width of 65 nm and a thickness of 20 nm.

### 3.7. Measurement of graphene ribbons with four electrical contacts grown by Co Cryo-FIBID

**Fig. 7:**
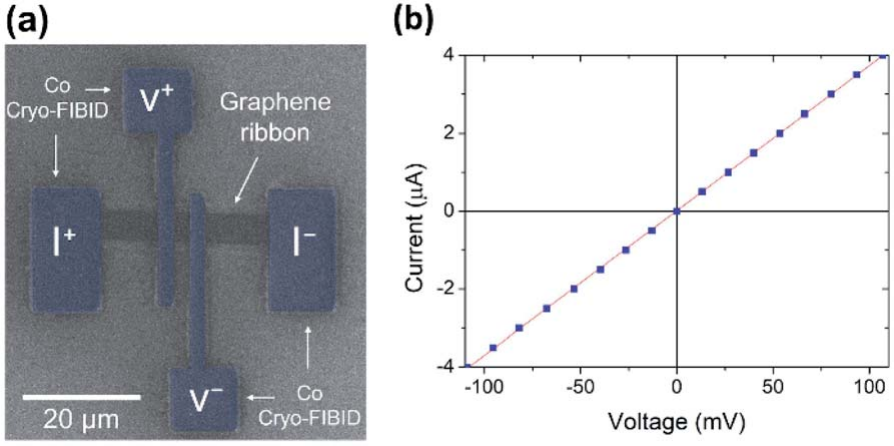
(a) Artificially coloured SEM micrograph of the measured graphene ribbon with four electrical contacts grown by Co Cryo-FIBID technique. (b) Current-*versus*-voltage dependence of the graphene ribbon, indicating a sheet resistance of 48 kΩ.

Given the low processing time, the minimized ion-induced damage, and the low electrical resistivity of the optimized Co Cryo-FIBID deposits, these can be used as metallic contacts on different materials: nanowires, 2D materials, or thin films of topological insulators, among others. In this work, we demonstrate for the first time their use as metallic contacts onto graphene ribbons (see Fig. 7a), following the steps described in Fig. 1. For this, we employed CVD graphene on Si/SiO_2_ substrate patterned into stripes by optical lithography and controlled oxygen plasma etching.

The metallic structures were grown under the optimized dose of 15 μC cm^−2^ and a beam current of 1 pA, which corresponds to less than 2 minutes of irradiation time. Therefore, in addition to reducing the ion irradiation dose 2 × 10^3^ times compared with Co-FIBID process at room temperature, we also eliminate the perturbing effects of Ga implantation and induced damage observed when the ion doses are higher. Four-probe *I*–*V* measurements performed on graphene ribbons are shown in Fig. 7b. The *I*–*V* curves exhibit a linear behaviour, with sheet resistance (*R*_□_) of 48 kΩ. This value agrees with previous measurements in the kΩ range obtained for large-scale graphene contacted with other metals like Pd (ref. 31) and Cu.^32^ The *R*_□_ is relatively higher than the values obtained with metal contacts fabricated by electron beam lithography,^33^ which can be attributed to possible surface charge doping near the contacts. Such doping can be controlled through current induced annealing^34^ or by covering graphene with other 2D materials such as hexagonal boron nitride.^35–37^ Overall, our electrical experiments provide a clear proof-of-concept that Cryo-FIBID can be successfully applied to fabricate metallic electrodes directly on 2D materials, despite their atomic-level thickness, which is considered to be tricky and prone to disorder creation.

### 3.8. Possibility of using Co Cryo-FIBID deposits as hard masks

**Fig. 8:**
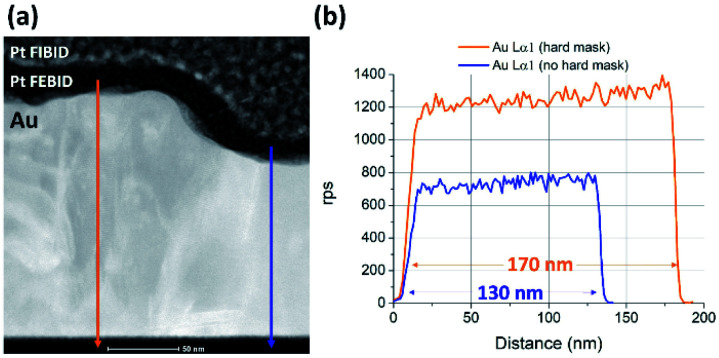
(a) High-resolution STEM image including the unprotected region on the right side and the protected region with Co Cryo-FIBID on the left side. (b) EDS profiles determining the Au thickness of both regions: the protected one in orange colour and the unprotected one in blue colour.

As a result of the nanometric lateral resolution in the growth of Co deposits by Cryo-FIBID, these are very promising for different applications in nanotechnology as a structural material. Moreover, the highly-efficient growth rate and the reduced ion-induced damage due to the low doses required are great advantages for these types of applications. As a proof of concept, we have used Co Cryo-FIBID deposits as hard mask on a 170 nm-thick gold layer. This experiment consisted in first growing two 30 nm-thick Co deposits in 10 × 5 μm^2^ areas that are separated by a distance of ∼300 nm and, secondly, carrying out a 2-min Ar^+^ milling process, with Ar^+^ ions accelerated under 250 V and current of 100 mA. In order to study how unprotected Au is removed by Ar^+^ milling compared to Au protected by Co Cryo-FIBID deposits, a lamella was extracted from an area that included both regions and studied by STEM, as shown in Fig. 8a. Two different EDS profiles were performed, on the protected region (orange colour) and the unprotected region (blue colour). The obtained results are shown in Fig. 8b, indicating that the thickness of unprotected gold was reduced by 40 nm and the protected gold kept the initial thickness.

Moreover, the compositional maps were performed by EDS in the protected region and results indicated that part of the Co Cryo-FIBID deposit is still present after ion milling (see Fig. S6 in ESI†). Thus, it can be concluded that Co Cryo-FIBID deposits can be successfully used to protect a 40 nm-thick Au layer from an Ar^+^ milling process. In some applications, the removal of the hard mask material with selectivity with respect to the underlying material is required. As far as we know, the etching of Co FIBID deposits has not been studied until now. However, one can assume that it could share similarities with standard Co etching by means of reactive ion etching^38^ or wet etching.^39^

## 4. Conclusions

The present work introduces for the first time the growth of nanopatterned cobalt deposits by Cryo-FIBID, further demonstrating its high potential for multiple applications. The key advantage of this cryogenic process is the ultra-fast growth rate and strikingly low charge dose required in comparison with FIBID and FEBID at room temperature. Co Cryo-FIBID deposits present electrical resistivities as low as 200 μΩ cm by using an optimized dose of 15 μC cm^−2^. Notably, both the resistivity value and the irradiation dose value are the lowest ones exhibited by any metallic deposit grown by Cryo-FIBID or Cryo-FEBID. Moreover, these cobalt deposits exhibit ferromagnetic behaviour with a magnetization value of 400 emu cm^−3^. Due to the low electrical resistivities, the minimized ion damage and the high resolution achieved, the fabrication of electrical contacts on two-dimensional materials becomes a relevant application. As a proof of concept, a graphene ribbon was successfully contacted and linear output characteristics with reasonable sheet resistance were observed. Finally, the potential of Co Cryo-FIBID for hard masking has also been demonstrated.

## Author contributions

A. S-P. has contributed to the investigation, data curation and analysis, and the writing of the original draft. S. S. has contributed to the investigation, data curation and analysis, and the writing (review and editing). C. M. has contributed to the conceptualization, the investigation, data curation and analysis, and the writing (review and editing). M. B. has contributed to the investigation and the writing (review and editing). P. P. has contributed to the investigation, data curation and analysis, and the writing (review and editing). D. B. and M. V. K. have contributed to the investigation and the writing (review and editing). P. C. has contributed to the supervision, the investigation and the writing (review and editing). J. M. D. T. has contributed to the conceptualization, supervision, the investigation, data curation and analysis, and the writing of the original draft.

## Conflicts of interest

There are no conflicts to declare.

## Supplementary Material

NA-003-D1NA00580D-s001
