# Supplementary material for: Highly-efficient growth of cobalt nanostructures using focused ion beam induced deposition under cryogenic conditions: application to electrical contacts on graphene, magnetism and hard masking
Source: Nanoscale Adv. 2021 Aug 25;3(19):5656–62. doi: 10.1039/d1na00580d (PMC9418482; doi:10.1039/d1na00580d)
Supplement: NA-003-D1NA00580D-s001 [file NA-003-D1NA00580D-s001.pdf]

Electronic Supplementary Material (ESI) for Nanoscale Advances.
This journal is © The Royal Society of Chemistry 2021

Supporting Information for

Highly-efficient growth of cobalt nanostructures using focused ion beam induced deposition under cryogenic conditions: application to electrical contacts on graphene, magnetism and hard masking

Alba Salvador-Porroche,[a] Soraya Sangiao,[a,b] César Magén,[a,b] Mariano Barrado,[b] Patrick Philipp,[c] Daria Belotcerkovtceva,[d] Venkata Kamalakar,[d] Pilar Cea,[a,b] José M. De Teresa,*[a,b]

[a] Instituto de Nanociencia y Materiales de Aragón (INMA), CSIC-Universidad de Zaragoza, 50009 Zaragoza, Spain. E-mail: deteresa@unizar.es; j.deteresa@csic.es

[b] Laboratorio de Microscopías Avanzadas (LMA), Universidad de Zaragoza, 50018 Zaragoza, Spain.

[c] Advanced Instrumentation for Nano-Analytics (AINA), MRT Department, Luxembourg Institute of Science and Technology (LIST), 41 rue du Brill, 4422 Belvaux, Luxembourg.

[d] Department of Physics and Astronomy, Uppsala University, Box 516, SE-751 20 Uppsala, Sweden.

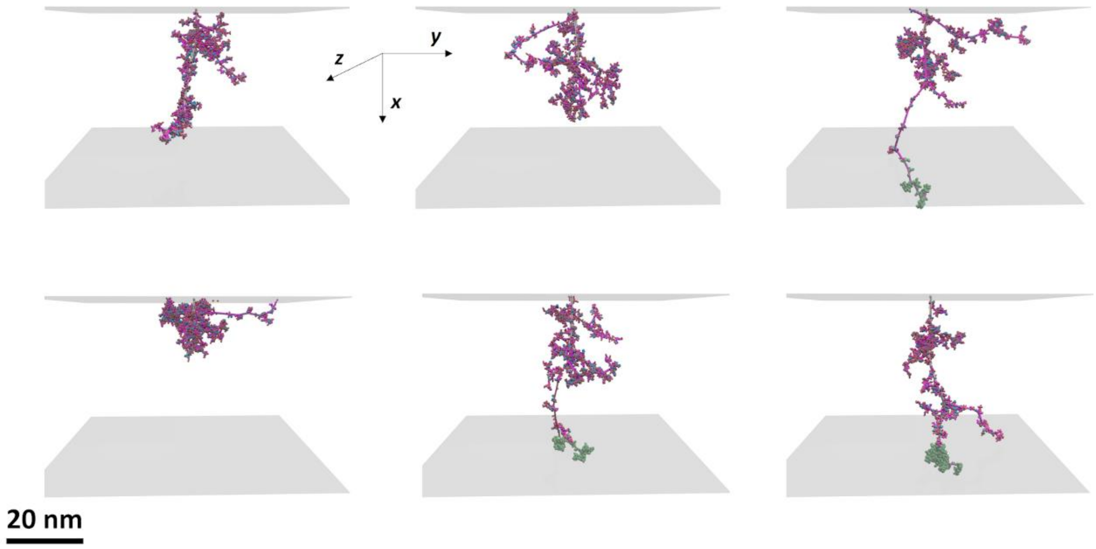

Fig. S1: Six different scenarios of collision cascades obtained by SDTRIMSP for 30 keV Ga^+ irradiation on a 44 nm-thick $Co_2(CO)_8$ condensed layer on top of a Si substrate. Colors of the species: Ga in grey, Si in green, Co in blue, C in magenta, O in red.

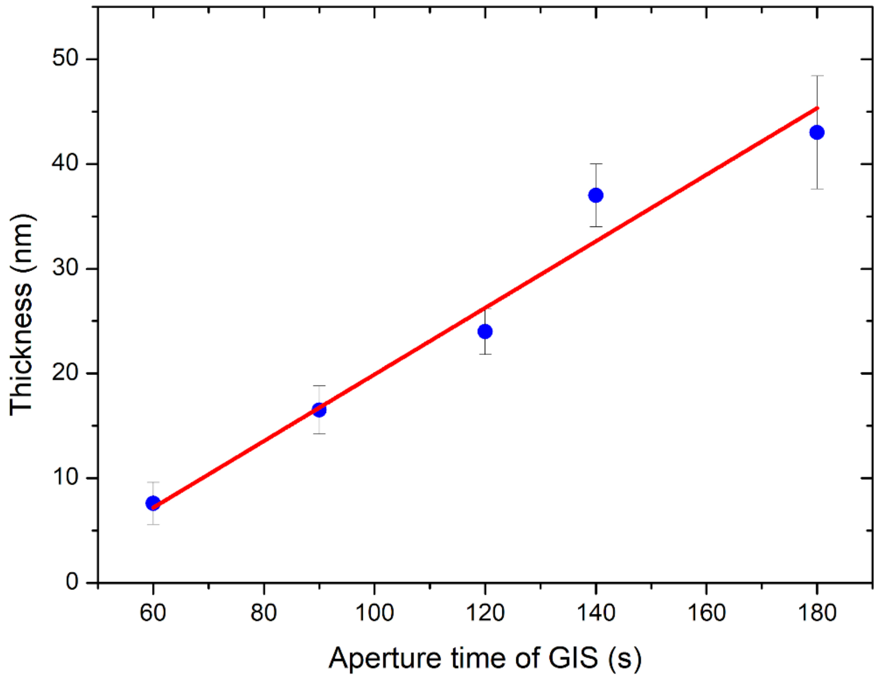

Fig. S2: Linear dependence between the thickness of the deposits after irradiation and the aperture time of the gas injector system (GIS).

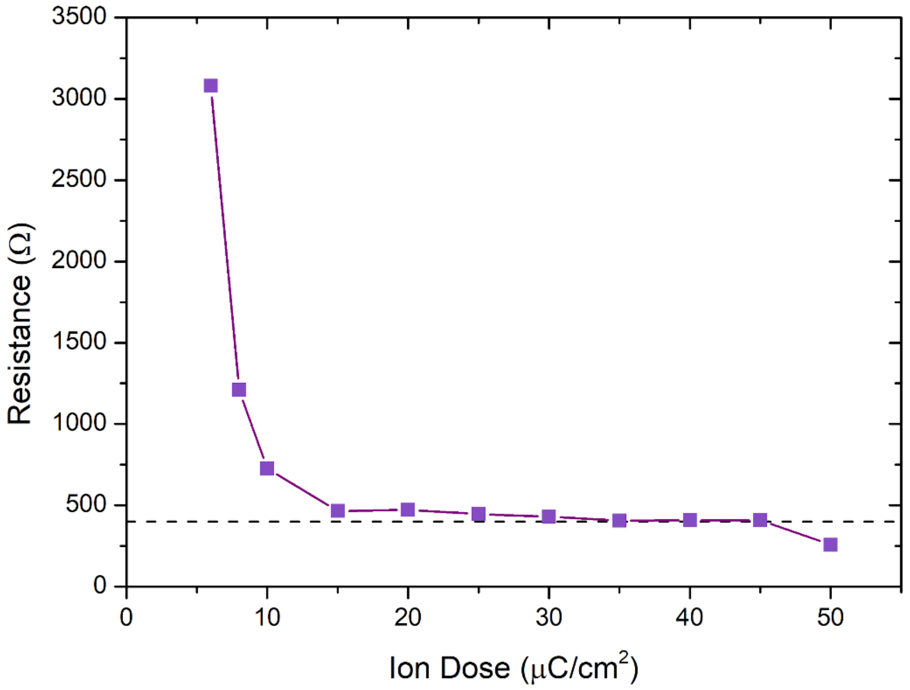

Fig. S3: Electrical resistance of cobalt deposits as a function of the ion dose for their fabrication by Cryo-FIBID technique. The lower resistance of the deposit grown with 50 μC/cm^2 is due to the smaller distance between the voltage contacts.

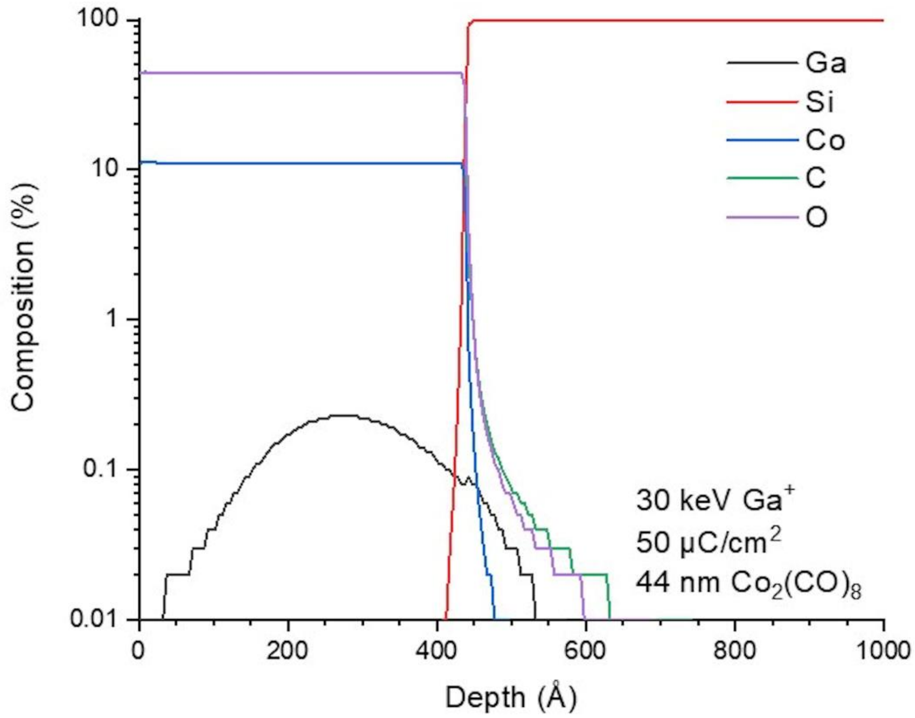

Fig. S4: Atomic composition of the $Co_2(CO)_8$ precursor layer versus depth obtained by SDTrimSP simulations for irradiation of 30 keV Ga^+ with an ion dose of 50 μC/cm^2. The emission of volatile species as CO_x is underestimated in these simulations.

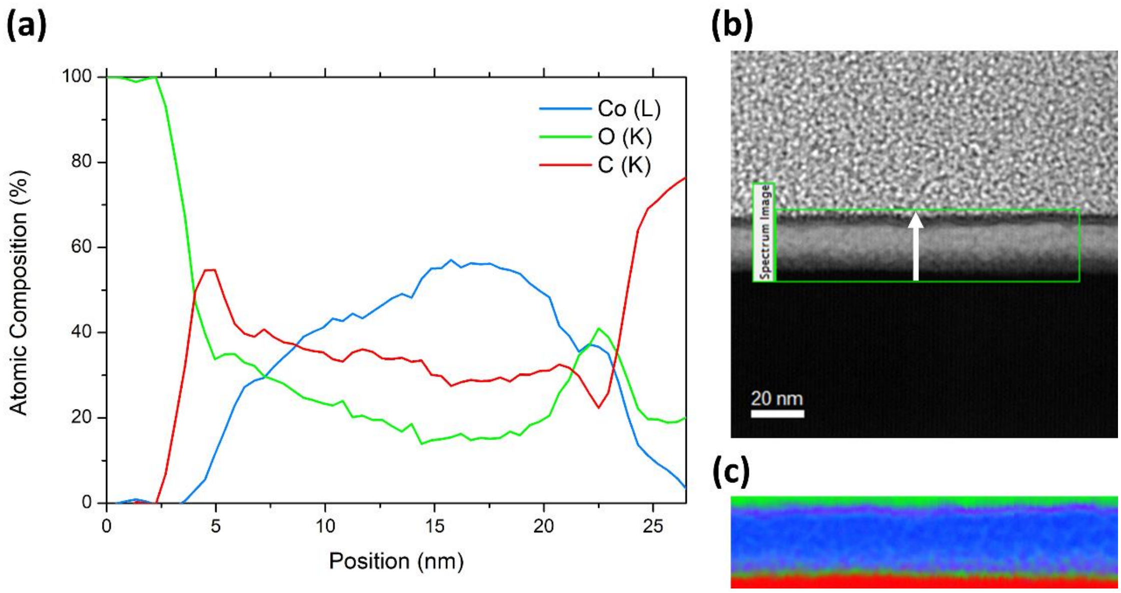

Fig. S5: (a) STEM-EELS chemical analysis along the cross-section of the 20 nm-thick deposit grown with an ion dose of 15 μC/cm^2. This profile is shown in the HAADF-STEM image in (b). (c) EELS chemical map of the elements that are present in our sample: carbon in red, oxygen in green and cobalt in blue.

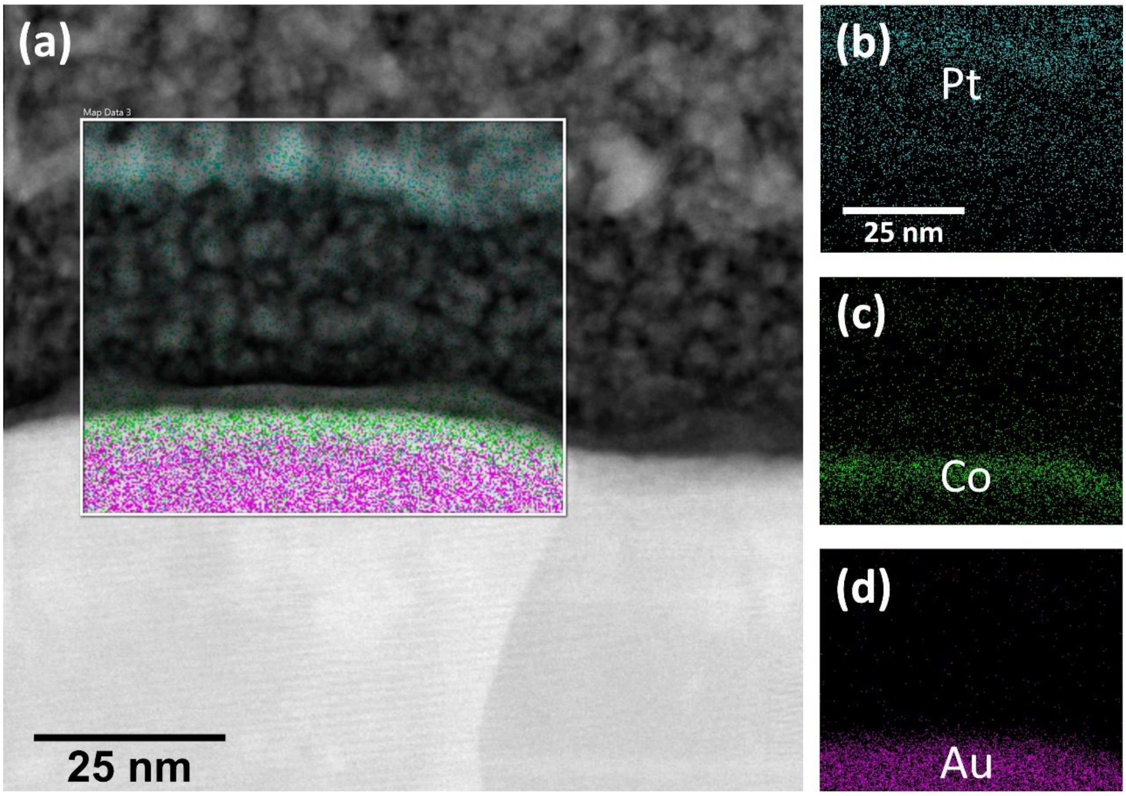

Fig. S6: STEM-EDS analysis in the cross-section of protected gold with Co Cryo-FIBID deposit. (a) Two dimensional EDS real density chemical map of the significant elements (b) Pt, (c) Co, and (d) Au.
